# p53‐dependent transcriptional suppression of BAG3 protects cells against metabolic stress via facilitation of p53 accumulation

**DOI:** 10.1111/jcmm.14764

**Published:** 2019-10-28

**Authors:** Jia‐Mei Wang, Bao‐Qin Liu, Zhen‐Xian Du, Chao Li, Jia Sun, Jing Yan, Jing‐Yi Jiang, Hua‐Qin Wang

**Affiliations:** ^1^ Department of Biochemistry & Molecular Biology China Medical University Shenyang China; ^2^ Key Laboratory of Cell Biology Ministry of Public Health Key Laboratory of Medical Cell Biology Ministry of Education China Medical University Shenyang China; ^3^ Department of Laboratory Medicine The 1st affiliated Hospital China Medical University Shenyang China; ^4^ Department of Endocrinology and Metabolism The 1st affiliated Hospital China Medical University Shenyang China

**Keywords:** BAG3, glucose insufficiency, metabolic stress, p53

## Abstract

Solid tumour frequently undergoes metabolic stress during tumour development because of inadequate blood supply and the high nutrient expenditure. p53 is activated by glucose limitation and maintains cell survival via triggering metabolic checkpoint. However, the exact downstream contributors are not completely identified. BAG3 is a cochaperone with multiple cellular functions and is implicated in metabolic reprogramming of pancreatic cancer cells. The current study demonstrated that glucose limitation transcriptionally suppressed BAG3 expression in a p53‐dependent manner. Importantly, hinderance of its down‐regulation compromised cellular adaptation to metabolic stress triggered by glucose insufficiency, supporting that BAG3 might be one of p53 downstream contributors for cellular adaptation to metabolic stress. Our data showed that ectopic BAG3 expression suppressed p53 accumulation via direct interaction under metabolic stress. Thereby, the current study highlights the significance of p53‐mediated BAG3 suppression in cellular adaptation to metabolic stress via facilitating p53 accumulation.

## 1 INTRODUCTION

Cells adjust their proliferation according to their availability of extracellular nutrients, which support energy generation and macromolecular synthesis necessary to undergo division. It is reported that glucose concentrations in tumour tissues are usually three to 10‐fold lower than in peripheral normal tissues ,^1^ which is possibly due to poor vascularization in tumour tissues, and more glucose consumption by cancer cells. Under nutrient limiting conditions, cancer cells must modulate their metabolism to adapt to the adverse circumstance. A fundamental concern in cancer biology is how cancer cells adjust to metabolically adverse conditions and whether this character of cancer cells can be exploited for therapeutic benefit.^2^, ^3^

Although the tumour suppressor p53 is best known for its key roles in the control of cell cycle and DNA damage response (DDR), accumulating evidences reveal that p53 also regulates diverse cellular metabolic processes.^4^ Loss of p53 permits unchecked cell cycle progression despite glucose limitation, but finally leads to decrease in cell viability.^5^, ^6^ Underlying these changes, p53 integrates a set of transcriptional processes that result in diverse cellular outcomes. However, the exact downstream contributors underlying p53‐mediated cell survival upon metabolic stress are not fully explored.

BAG3 is one member of the human BAG cochaperone family (BAG1‐6) that interacts with the ATPase domain of the heat shock protein 70 (HSP70) via conserved BAG domain.^7^, ^8^ BAG3 exhibits a wide range of biological functions including the regulation of stress responses, autophagy, cellular survival, apoptosis and viral replication.^9^, ^10^ BAG3 is constitutively expressed in cardiac myocytes and skeletal muscle cells, but is induced by different stimuli in many other cell types.^9^, ^11^, ^12^, ^13^, ^14^, ^15^ Inducible BAG3 expression usually serves as a cellular protective mechanism against stressful stimuli.^7^, ^16^, ^17^, ^18^, ^19^, ^20^, ^21^ Recently, we have demonstrated that BAG3 promotes aerobic glycolysis and growth of pancreatic cancer cells by stabilizing HK2 mRNA via interaction with HK2 transcripts,^22^ assigning BAG3 another functions to RNA binding and metabolic reprogramming.

In the present study, we show that BAG3 is suppressed in a p53‐dependent pattern under metabolic stress triggered by glucose limitation. Ectopic BAG3 expression inhibits p53 accumulation via direct interaction and compromises cellular adaptation to glucose limitation. To our knowledge, the current study for the first time demonstrates that p53‐mediated suppression of BAG3 facilitates p53 accumulation and cellular survival upon glucose insufficiency‐induced metabolic stress. These results significantly enhance our understanding on the molecular mechanism(s) by which cells adapt to metabolic stress. Moreover, these insights provide a novel therapeutic target to alter metabolic activity and selectively eliminate cancer cells.

## 2 MATERIALS AND METHODS

### 2.1 Cell line cultivation

HCT116, Bel‐7402 and MCF7 cells were maintained in DMEM (Biological Industries, BI) that was supplemented with 10% foetal bovine serum (Biological Industries, BI).

### 2.2 Chromatin immunoprecipitation (ChIP)

Chromatin immunoprecipitation assays were performed using a kit from Upstate Biotechnology Inc The cells were fixed with 1% formaldehyde to cross‐link chromatin according to the manufacturer's instructions. The cell lysates were prepared and sonicated on ice to break chromatin DNA to an average length of 400 bp. After a pre‐clearing step, immunoprecipitation was carried out at 4°C overnight with anti‐p53 antibody or normal goat IgG (negative control antibody). Immune complexes were collected with salmon sperm DNA saturated protein A‐agarose beads. After extensive washing, the immunoprecipitated complexes were eluted with 0.1 mol/L NaHCO_3_ and 1% SDS. The protein‐DNA cross‐links were then reversed by incubating at 65°C for 5 hours. DNA was subsequently purified using a proteinase K digestion with a phenol: chloroform extraction and an ethanol precipitation. Real‐time PCR was performed using PCR primers specific for the BAG3(GI: 9531) RE1 sequence between nucleotide positions −29885 and −29876 including the forward primer 5'‐GTGATTCTCCCACCTCAGCCTTCT‐3' and the reverse primer 5'‐GGCCAATGTAGTGAAACCCCGTCT‐3'. PCR amplification resulted in a 110 bp product that contained the BAG3 RE1 sequence. In addition, PCR amplification was conducted to amplify the RE2 sequence between nucleotide positions +5143 and +5152 using the forward primer 5'‐ACAGGCTAAAAGTGGGTTGGA‐3' and the reverse primer 5'‐ TGTGGCCCAGAAGCCACTTA‐3'), which generated an 85 bp product. In addition, the p21 sequence was used as a positive control by amplifying the sequence between −2421 and −2412 positions using the forward primer 5'‐CTGAGCCTCCCTCCATCC‐3' and the reverse primer 5'‐GAGGTC TCCTGTCTCCTACCA TC‐3', which generated a 189 bp amplification product. Standard curves were then calculated from the amplification data. The abundances of the BAG3 promoter that were amplified were considered as the IP and Input. In order to facilitate comparison, amplification results were expressed as IP/Input ratios of PCR products.

### 2.3 Label and capture nascent RNA

Nascent synthesized RNA was labelled and captured using the Click‐iT Nascent RNA Capture kit (Thermo Fisher Scientific). Cells were pulsed with 5‐ethynyl uridine for 4 hours. Total RNA was isolated and subjected to nascent RNA capture and analysed by real‐time PCR.

### 2.4 Cell proliferation assay

Cells were seeded into six‐well plates at a density of 100 000 cells per well, 2 mL of medium supplement 10% FBS per well. Change the medium every 24 hours. The cells were incubated with trypan blue solution at 1:1. Cell number was determined by counting with automated cell count kit.

### 2.5 Detection of apoptotic cell death

To detect apoptotic cell death, cells were washed twice with PBS and then stained using the Annexin V/PI Apoptosis Detection Kit according to the manufacturer's instructions. After staining with Annexin V and PI for 30 minutes, samples were analysed using a fluorescence‐activated cell scanner flow cytometer.

### 2.6 Western blot and immunoprecipitation

Cells were cultured and harvested followed by lysing in buffer containing 20 mmol/L Tris‐HCl, 150 mmol/L NaCl, 2 mmol/L EDTA, 1% Triton‐X100 and freshly added protease and phosphatase inhibitors. Equivalent amounts of protein were subjected to SDS‐PAGE under reducing conditions in 10% SDS‐PAGE and then transferred to PVDF membranes. Cell lysates were pre‐cleared with protein A/G magnetic beads for immunoprecipitation, and the protein A/G magnetic beads were treated with various antibodies followed by incubation overnight at 4°C. The immunoprecipitants were washed with lysis buffer three times and analysed by Western blot analysis, which was performed using primary antibodies against Flag, Myc antibodies. The following antibodies were used in this study: BAG3 (GeneTex); p53 (DO‐1, Santa Cruz, 49‐1031, Thermo Fisher); Flag (Cell Signalling Technology); and p62, GAPDH (Millipore).

### 2.7 Crystal Violet Assay

To visualize cells with crystal violet, cells were seeded into 12‐well plates at a density of 50 000 cells per well along with 1 mL of media that was supplemented with 10% FBS and 1 mmol/L glucose, followed by incubation for 16 hours. After complete washing with PBS, cells were incubated with crystal violet solution for 30 minutes at room temperature. The stain was solubilized in 33% acetic acid, and staining was evaluated at 570 nm.

### 2.8 Cell Cycle Analysis

To investigate alteration of cell cycles, cells in logarithmic growth phase were seeded into six‐well plates and maintained in FBS‐free medium for 12 hours. The cells were then fixed in 70% ethanol over 18 hours. The fixed cells were stained with a 50 μg/mL propidium iodide (PI) solution and Rnase A for 1 hour. The fluorescence was measured using the Becton Dickinson FACScan. Cell Cycle phase analysed by ModFIT software.

### 2.9 In situ proximity ligation assay

In situ proximity ligation assays (PLA) were conducted using the Duolink PLA kit (Sigma‐Aldrich) according to the manufacturer's protocol. For these analyses, cells were seeded into a chamber slide at a concentration of 5000 cells per well. PLA probes were diluted in the appropriate buffer at a ratio of 1:5 and added to the sample, incubated 1 hour at 37°C. Ligation, dilute the 5x Ligation buffer 1:5 in high purity water and mix for 1 hour and wash the slides 5 minutes in 1x Wash Buffer A at room temperature. Amplification, tap off the ligation solution from the slides, add polymerase to the 1x amplification buffer at a 1:80 dilution and mix. Incubate the slides in a pre‐heated humidity chamber for 100 minutes at 37°C. Fluorescence was visualized under a confocal microscopy, using at least a 60x objective.

### 2.10 Isolation and culture of MEF cells

Pregnant mice at 13 or 14 days post‐coitum were sacrificed by cervical dislocation and the limbs, heads and bones were removed for dissected genotyping. Recovered tissues were then transferred to a new petri dish containing cold PBS, and the remaining tissues were incubated with trypsin containing DNase I. Isolated cells were then transferred to Collagenase IV for 1 hour. Place the 200 mesh screen in a new dish and drop the cell suspension onto the screen for filtration. Primary MEF cells can attach to the walls of flasks. Consequently, primary MEF cells were maintained in DMEM that was supplemented with 10% foetal bovine serum and incubated at 37°C with 5% CO_2_.

### 2.11 Statistical analyses

The statistical significance of differences among data was analysed with an analysis of variance (ANOVA) and post hoc Dunnett's tests. Statistical significance was defined as *P* < .05. All experiments were repeated in triplicate, and the data are expressed as the mean ± SD (standard deviation).

## 3 RESULTS

### 3.1 Glucose insufficiency specifically decreases BAG3 expression in a p53‐dependent manner

**Figure 1:**
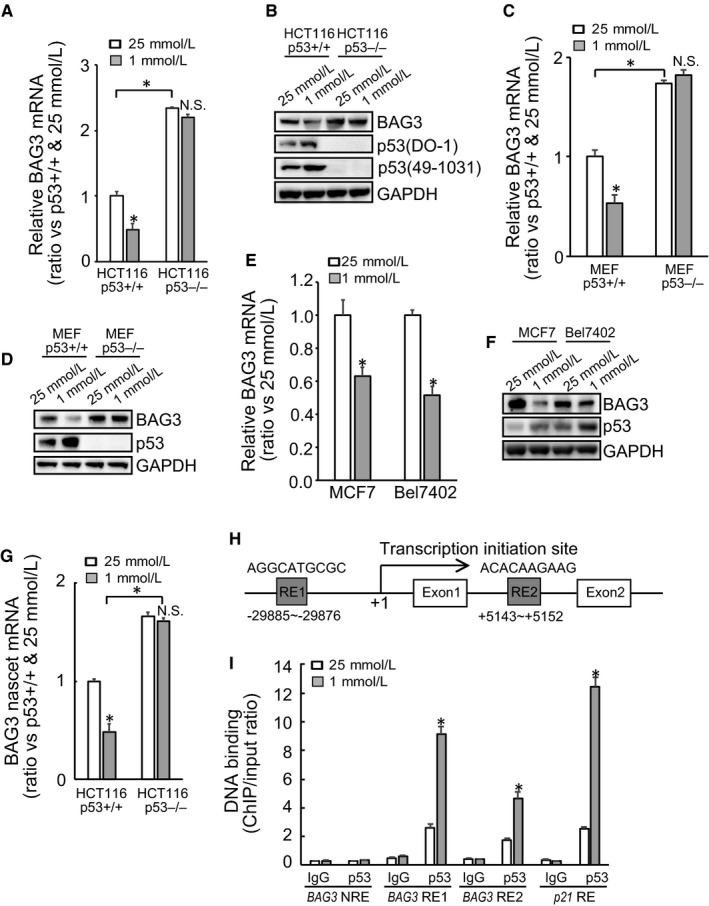
Glucose limitation suppresses BAG3 expression in dependent of p53. (A‐B), real‐time RT‐PCR analysis of BAG3 mRNA (A) and immunoblot analysis of BAG3 protein (B) in HCT116 cells with wild‐type p53 (p53+/+) or deletion (p53−/−). (C‐D), real‐time RT‐PCR analysis of BAG3 mRNA (C) and immunoblot analysis of BAG3 protein (D) in MEF p53+/+ and p53−/− cells. E‐F, real‐time RT‐PCR analysis of BAG3 mRNA (E) and immunoblot analysis of BAG3 protein (F) in MCF7 and Bel‐7402 cells. G, Analysis of nascent BAG3 mRNA in HCT116 p53+/+ and p53−/− cells. H, Schematic diagram of the BAG3 gene with two potential p53 responsive element (RE). I, ChIP analysis of p53 recruitment to the indicated DNA

The activity of p53 is critical for cell survival upon metabolic stress induced by lack of glucose. However, the exact its downstream effectors remained largely unknown. Real‐time PCR shows that BAG3 was significantly decreased by extremely low glucose (1 mmol/L) in HCT116 cells with wild‐type p53 (p53+/+), while its expression was unaltered in HCT116 cells with p53 deletion (p53−/−) (Figure 1A). Consistent with mRNA expression, Western blot demonstrated that 1mM glucose culture decreased BAG3 expression in p53+/+ HCT116 cells, while unaltered BAG3 expression in p53−/− HCT116 cells (Figure 1B). It should be noted that compared with its control partner, p53 null HCT116 cells exhibited higher BAG3 expression levels under high glucose culture condition (Figure 1A,B). Similar like in HCT116 cells, low glucose decreased BAG3 mRNA (Figure 1C) and protein (Figure 1D) in p53+/+ MEF cells. In addition, deletion of p53 increased BAG3 expression in MEF cells, which was unaltered by low glucose (Figure 1C,D). Low glucose also decreased BAG3 mRNA (Figure 1E) and protein (Figure 1F) expression levels in Bel‐7402 and MCF7 cells, both of which contain wild‐type p53. Nascent RNA analysis demonstrated that glucose insufficiency significantly decreased novel synthesis of BAG3 mRNA in HCT116 cells with p53 (Figure 1G). These data indicated that BAG3 might be suppressed by glucose insufficiency in a p53‐dependent manner. Searching on BAG3 gene found two potential p53 responsive elements (RE1 and RE2) located on distant upstream and intron 1 of BAG3 gene, respectively (Figure 1H). ChIP was then performed and demonstrated that p53 was recruited to both RE1 and RE2 of BAG3 gene, which was increased by glucose insufficiency (Figure 1I). As a positive control, p53 was recruited to p21 promoter, which was augmented by glucose insufficiency (Figure 1I). On the contrary, a fragment BAG3 intron that does not contain a potential p53 responsive element (NRE) was not immunoprecipitated by p53 (Figure 1I).

### 3.2 Glucose limitation had no effects on expression of other BAG members

**Figure 2:**
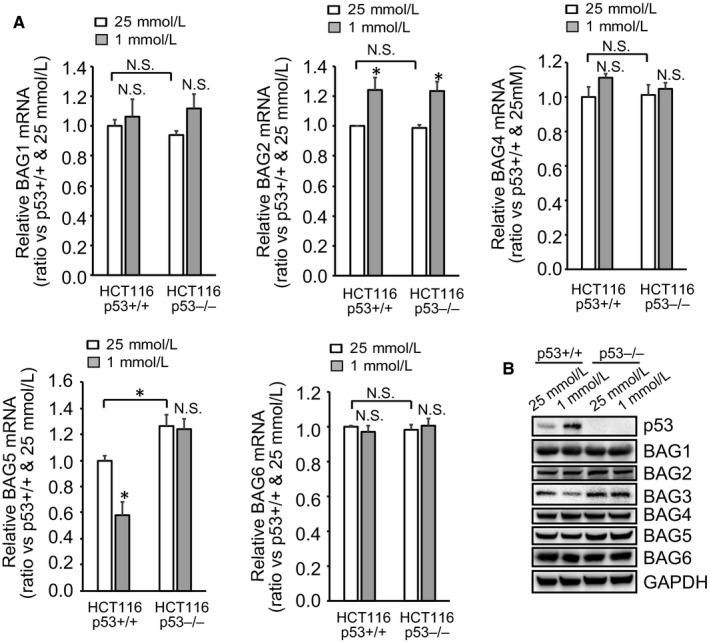
Glucose limitation had not effects on expression of other BAG members. (A‐B), real‐time RT‐PCR analysis of BAG members mRNA (A) and immunoblot analysis of BAG members protein in HCT116 cells

Glucose insufficiency resulted in similar pattern of alteration in BAG5 mRNA (Figure 2A). BAG1, BAG4 and BAG6 mRNA expression was unaltered, while BAG2 mRNA was increased irrespective of p53 status by glucose insufficiency (Figure 2A). Western blot demonstrated that other members of BAG family including BAG5 were unaltered by glucose insufficiency (Figure 2B). These data indicated that BAG3 was specifically suppressed by glucose insufficiency.

### 3.3 BAG3 reduction is a protective response of cells to glucose insufficiency

**Figure 3:**
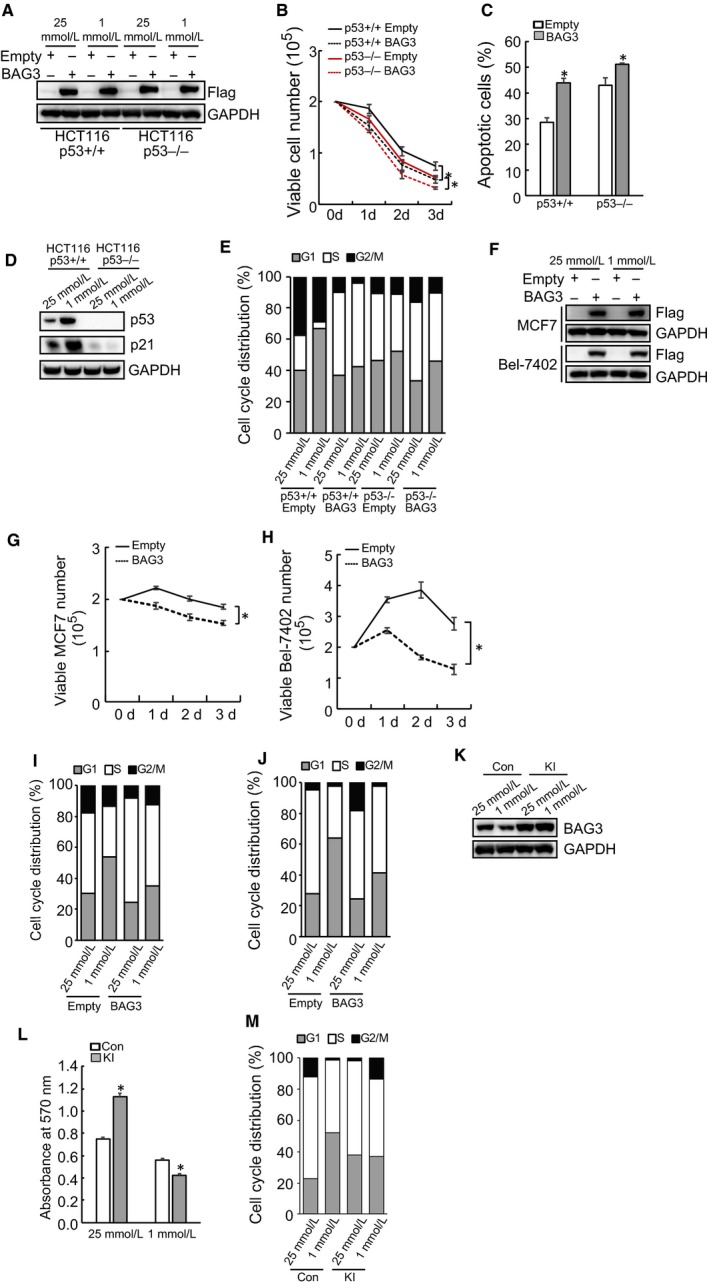
BAG3 reduction is a protective response of cells to glucose insufficiency. A, immunoblot analysis of ectopic BAG3 in HCT116 cells infected with lentivirus containing empty or BAG3 construct. B, viable cell count of HCT116 cells cultured under 1mM glucose. C, apoptotic cell analysis of HCT116 cells. D, immunoblot analyses of p53 and p21 protein in HCT116 cells. E, cell cycle analysis of HCT116 cells. F, immunoblot analysis of ectopic BAG3 in MCF7 and Bel‐7402 cells. (G‐H), viable cell count of MCF7 (G) and Bel‐7402 (H) cells cultured under 1mM glucose. (I‐J), cell cycle analysis of MCF7 (I) and Bel‐7402 (J) cells. K, immunoblot analysis of BAG3 expression in control or BAG3 knock in (KI) MEF cells. L, crystal violet assay for MEF cells. M, cell cycle analysis of MEF cells

To investigate the possible function of BAG3 down‐regulation in response to glucose insufficiency, BAG3 was ectopically introduced to HCT116 cells (Figure 3A). Forced BAG3 expression significantly decreased viable HCT116 cell numbers irrespective of p53 status under low glucose culture (Figure 3B). Flowcytometry demonstrated that ectopic BAG3 expression increased apoptosis of HCT116 cells under low glucose culture irrespective of p53 status (Figure 3C), but the only small effect is observed in p53 KO cells when comparing empty to BAG3, whereas in p53 WT cells there is much more apoptosis in BAG3 comparing to empty condition, so the ectopic BAG3 increased apoptosis of HCT116 cells dependent or partial dependent p53. Cell cycle progression is sensitive to nutrient availability. p53 is activated and plays a critical role in the cell cycle arrest under low glucose culture. However, our understanding on how BAG3 intersects with cell cycle progression is incomplete. The glucose limitation activates p53 signalling, including p21 expression. Western blot proved that glucose limitation p53‐dependently induced p21 in HCT116 cells (Figure 3D). Cell cycle analyses demonstrated that glucose insufficiency blocked p53+/+ HCT116 cells at G1 phase significantly, while cells with p53 deletion or ectopic BAG3 expression continued cell cycle progression despite lack of glucose, leading to a rapid decline in cell viability.(Figure 3E). BAG3 was ectopically introduced to MCF7 and Bel‐7402 cells (Figure 3F). Ectopic BAG3 expression decreased viability of MCF7 (Figure 3G) and Bel‐7402 (Figure 3H) cells cultured under extremely low glucose condition. In addition, ectopic BAG3 expression compromised G1 arrest triggered by glucose insufficiency in both MCF7 (Figure 3I) and Bel‐7402 (Figure 3J) cells. MEF cells were then separated from the control or BAG3 knock‐in (KI) embryos (Figure 3K). BAG3 increased proliferation of MEF cells under high glucose culture, while decreased MEF cell viability upon glucose insufficiency (Figure 3L). In addition, glucose insufficiency did not trigger cell cycle arrest in MEF cells with BAG3 KI (Figure 3M).

### 3.4 BAG3 decreases p53 activity under glucose insufficiency

**Figure 4:**
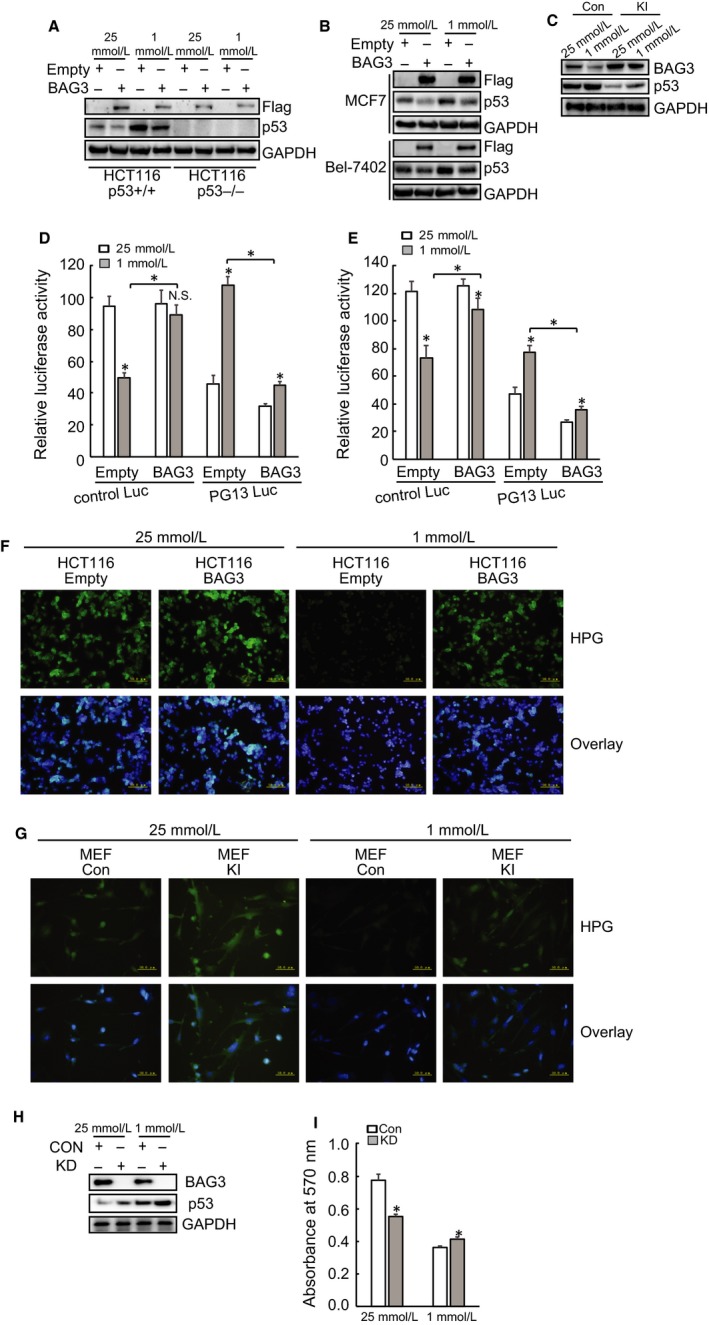
BAG3 decreases p53 activity under glucose insufficiency. (A‐C), immunoblot analysis of p53 expression in HCT116 (A), MCF7 and Bel‐7402 (B), and MEF (C) cells. D‐E, Luciferase assays for p53 responsive construct in HCT116 (D) or MEF (E) cells. (F‐G), Analysis of novel protein synthesis by HPG incorporation in HCT116 (F) or MEF (G) cells. H, immunoblot analysis of BAG3 expression in HCT116 cells. I, crystal violet assay for HCT116 cells

Ectopic BAG3 decreased p53 expression under both high and extremely low glucose culture in HCT 116 (Figure 4A), MCF7 and Bel‐7402 (Figure 4B), as well as MEF (Figure 4C) cells. Consistent with p53 expression, luciferase activity assays demonstrated that glucose insufficiency significantly increased the luciferase activity of pGL13 Luc construct, which contains p53 responsive element, in HCT116 cells (Figure 4D). Ectopic BAG3 expression significantly decreased the luciferase activity of pGL13 Luc under glucose insufficiency (Figure 3D). The luciferase activity of control Luc construct was significantly decreased by glucose insufficiency in control HCT116 cells, while it was rarely affected in cells with ectopic BAG3 expression (Figure 4D). Similar regulation of control and pGL13 Luc activities by ectopic BAG3 expression was observed in MEF cells (Figure 4E).

To explore the potential mechanisms underlying maintenance of control Luc expression under glucose insufficiency by ectopic BAG3 expression, novel protein synthesis was analysed using HPG and demonstrated that glucose insufficiency significantly blocked protein synthesis in control HCT116 (Figure 4F) and MEF (Figure 4G) cells. On the other hand, HCT116 (Figure 4F) and MEF (Figure 4G) cells with BAG3 overexpression demonstrated active protein synthesis under glucose insufficiency. BAG3 knockdown increased p53 expression in HCT116 cells (Figure 4H). BAG3 knockdown suppressed proliferation of HCT116 cells under high glucose culture, while increased cell viability upon glucose insufficiency (Figure 4I).

### 3.5 BAG3 promotes calpain‐dependent p53 degradation under metabolic stress

**Figure 5:**
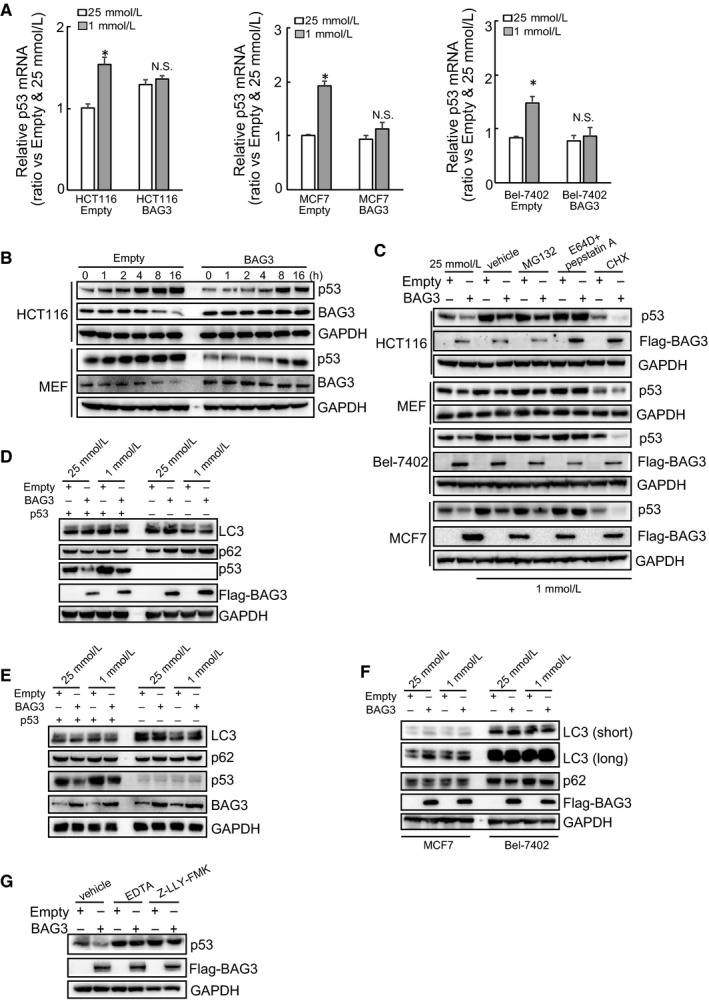
BAG3 promotes calpain‐dependent p53 degradation under metabolic stress. A, real‐time RT‐PCR analysis of p53 mRNA in HCT116, MCF7 and Bel‐7402 cells. B, immunoblot analysis of p53 in HCT116 and MEF cells cultured with 1 mmol/L glucose for the indicated time. C immunoblot analysis of p53 in the presence of protein degradation inhibitors (MG132, E64D and pepstatin A) or protein synthesis inhibitor cycloheximide (CHX) in the indicated cells. (D‐F), HCT116 (D), MEF (E), MCF7 and Bel‐7402 (F) cells were treated with 25 and 1 mmol/L glucose, immunoblot analyses were performed using the indicated antibodies. G, immunoblot analysis of p53 in HCT116 cells cultured under 1mM glucose in the presence of calpain inhibitor EDTA and Z‐LLY‐FMK

Real‐time PCR demonstrated that no unanimous regulation of p53 mRNA was observed by glucose limitation in HCT116 cells, as well as Bel‐7401 and MCF7 cells (Figure 5A). These data indicated that glucose insufficiency may induce p53 expression mainly at the protein level. Western blot demonstrated that p53 was effectively induced by glucose insufficiency at 4 and 1 hour in control HCT116 and MEF cells, respectively, which was delayed to 8h exposure in cells with ectopic BAG3 expression (Figure 5B). At p53 was significantly induced at 8 hours in both control and ectopic BAG3 expression cells, cells were then cultured with extremely low glucose for 8 hours in the presence of MG132 to block proteasomal and E64D/pepstatin A to block lysosomal degradation of p53, respectively, or cycloheximide (CHX) to block novel protein synthesis. CHX significantly suppressed p53 accumulation induced by glucose insufficiency in cells infected with both empty and BAG3 containing lentivirus (Figure 5C). Even in the presence of CHX, ectopic BAG3 expression decreased p53 levels (Figure 5C). MG132 demonstrated no effect, whereas E64D/pepstatin A completely blocked ectopic BAG3‐mediated p53 reduction in HCT116, MEF, MCF7 and Bel‐7402 cells (Figure 5C). Ectopic BAG3 demonstrated no constant effect on autophagy under glucose insufficiency in HCT116 (Figure 5D), MEF (Figure 5E), MCF7 and Bel‐7402 (Figure 5F) cells, excluding the possibility that BAG3 promoted non‐selective degradation of p53 by autophagy. It has been reported that p53 is degraded via calpain‐dependent manner under some circumstance,^22^ EDTA was then used to chelate Calcium to block calpain activity, and in addition, a specific calpain inhibitor Z‐LLY‐FMK was also used. Similar like E64D/pepstatin A, both EDTA and Z‐LLY‐FMK, significantly increased p53 expression in HCT116 cells with forced BAG3 expression cultured under 1mM Glucose (Figure 5G).

### 3.6 BAG3 compromises cellular adaption to metabolic stress via direct interaction with p53

**Figure 6:**
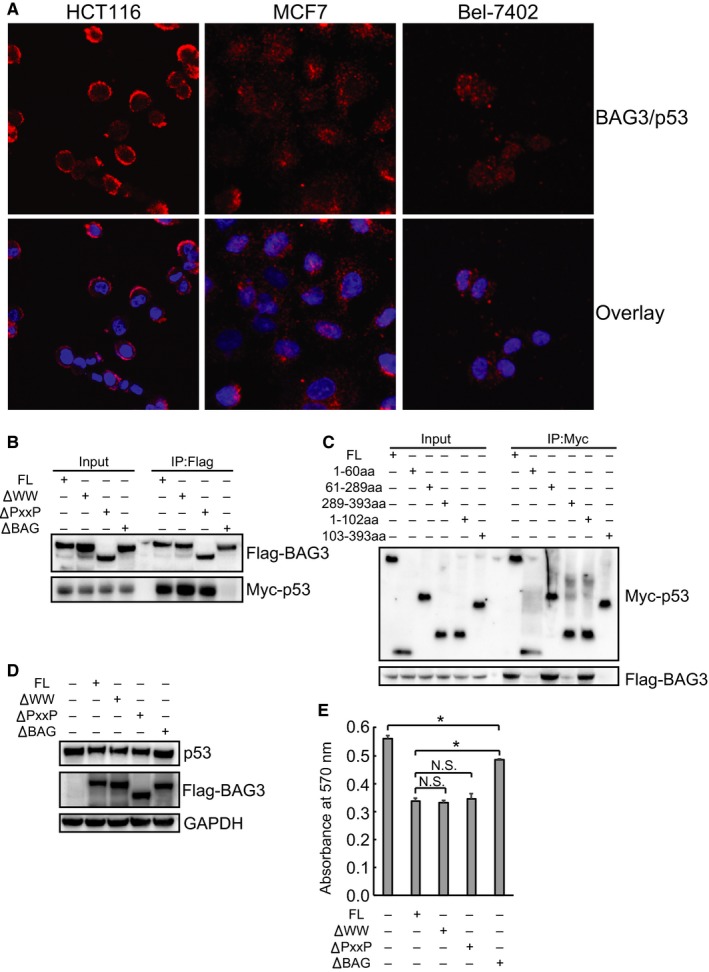
BAG3 suppresses cell viability under metabolic stress via direct interaction with p53. A, DuoLink PLA analysis of endogenous expression BAG3 and p53 interaction in HCT116, MCF7 and Bel‐7402 cells. B, immunoblot analysis from immunoprecipitated Flag‐tagged full‐length (FL) or mutant BAG3 in HEK293 cells. C, immunoblot analysis from immunoprecipitated Myc‐tagged FL or mutant p53 in HEK293 cells. D, immunoblot analysis of p53 in HCT116 cells transfected with FL or mutant BAG3. E, crystal violet assay for HCT116 cells transfected with FL or mutant BAG3

As global screen of BAG3 interactomes has identified p53 might be a potential binding partner of BAG3, we then explored whether BAG3 promoted calpain‐dependent p53 degradation via their interaction. DuoLink PLA confirmed that endogenous BAG3 directly interacted with p53 in HCT116, MCF7 and Bel‐7402 cells (Figure 6A). In addition, co‐IP using mutant BAG3 constructs without WW, PxxP or BAG domain demonstrated that full‐length (FL) BAG3 and mutant BAG3 without WW or PxxP domain coimmunoprecipitated with p53, while deletion of BAG domain blocked interaction of BAG3 with p53 (Figure 6B), indicating that BAG3 interacted with p53 through its BAG domain. Truncated p53 constructs were then generated, and co‐IP demonstrated that FL p53, p53 fragment with 61‐289aa, as well as p53 fragment with 1‐102aa coimmunoprecipitated with BAG3 (Figure 6C), indicating that p53 interacted with BAG3 through its 61‐102aa. To confirm whether BAG3 destabilized p53 via interaction, p53 expression was investigated in HCT116 cells were transfected with mutant BAG3 constructs. Consistent with co‐IP data (Figure 6B), similar like FL BAG3, mutant BAG3 without WW or PxxP domain also suppressed, while mutant BAG3 without BAG domain demonstrated no obvious effect on p53 accumulation triggered by glucose limitation (Figure 6D). Compared with FL BAG3, mutant BAG3 with WW or PxxP deletion exhibited similar extent of suppression on cell viability under glucose limitation for 16 hours (Figure 6E). On the other hand, deletion of BAG domain significantly rescued the suppressive effect mediated by BAG3 (Figure 6E). These data indicated that BAG3 suppressed cellular viability under metabolic stress at least partly by suppression of p53 accumulation via direct interaction. It should be noted that mutant BAG3 with BAG deletion still reduced cell viability under glucose limitation, although with some smaller degree (Figure 6E), indicating that alternative mechanism(s) except for p53 might underly regulation of cellular response to metabolic stress by BAG3.

## 4 DISCUSSION

Sufficient glucose supply is a mandatory requirement for execution of replicative cell division. Proliferating mammalian cells sense metabolic stress induced by short of nutrient supply, once glucose provision is insufficient, cell cycle checkpoint occurs at the G1/S boundary despite the sustained availability of amino acid substrates.^5^ The coordination of glucose availability with cell cycle transition permits cell to adjust to unfavourable microenvironments and represents a nutrient‐sensing pathway to suspend cell growth before nutrient availability falls below threshold that cannot sustain cell survival any more.^5^ Therefore, understanding signalling pathways underlying cellular adaptation to metabolic stress has emerged as a focus in the field of cancer.

In addition to its critical functions in the control of cell cycle and DNA damage response, the tumour suppressor protein p53 regulates a range of cellular metabolic procedures including glycolysis,^23^, ^24^ oxidative phosphorylation,^23^ pentose phosphate pathway (PPP),^25^ and glutaminolysis.^26^ In addition, activation of p53 by glucose limitation plays a critical role to suppress cell cycle progression.^5^ In contrast to its role in promoting apoptosis after DNA damage stress, p53 also contributes to cellular survival during metabolic stress that is induced by glucose limitation.^5^ p53 is well known for its function as a transcription factor, and its activation produces versatile biological consequences by coordinating various integrated transcriptional processes. Thereby, decoding the contributors underlying a specific function of p53 is a significant problem in the area of p53 research. However, the exact downstream molecules underlying its protective role during metabolic stress are not completely studied. The present study demonstrated that metabolic stress induced by glucose limitation suppressed BAG3 expression in HCT116 and MEF cells burden WT p53, while had no effect in p53 null HCT116 and MEF cells, indicating that glucose insufficiency might inhibit BAG3 expression in a WT p53‐dependent pattern. Two potential p53‐responsive elements were identified in BAG3 gene. In addition, the current study demonstrated that p53 was recruited to potential p53‐responsive elements located on the *BAG3* gene, which was enhanced by glucose insufficiency. These data verified BAG3 as a novel unreported p53‐responsive gene under metabolic stress induced by glucose limitation. However, as most cancer cells burden mutant p53, but not complete deletion of p53, whether mutant p53 could repress BAG3 expression upon glucose insufficiency requires further investigation in the future study.

BAG3 plays a far‐ranging regulatory function in apoptosis, development, cytoskeleton arrangement and autophagy.^9^, ^10^ Induction of BAG3 generally endows survival under stressful circumstance while its down‐regulation promotes apoptosis in a variety of cell models. Consequently, induction of BAG3 is considered as a protective anti‐stress response. However, counterintuitive for an envision of stress‐inducible and pro‐survival gene, the current study demonstrated that BAG3 was suppressed rather than induced by metabolic stress mediated by glucose limitation. In addition, hindrance of BAG3 down‐regulation dampened cell survival during glucose limitation, indicating that BAG3 down‐regulation downstream of p53 activation might be a protective mechanism underlying adaption of cells to metabolic stress induced by glucose insufficiency. Further investigations are needed to clarify whether BAG3 is responsive to p53 activation and suppressed by other stimuli, such as DNA damage, as well as the potential involvement of BAG3 regulation under such circumstances. In addition, the mechanism(s) underlying pro‐survival and anti‐survival function of BAG3 remains large unknown, which requires further investigation. BAG3 has a modular structure with multiple protein‐interacting domains. Thereby dynamic interaction with distinct sets of proteins might be responsible for its seemingly contradictory effect under different circumstances. Alternatively, post‐translational modification might also provide BAG3 with discrepant function. For example, phosphorylation of BAG3 at Ser178 promoted, while non‐phosphorylatable BAG3 mutant decreased migration and invasion of thyroid cancer cells.^27^

BAG3 interacts with diverse proteins, which enables it to participate in various biological and pathological pathways. The current study demonstrated that BAG3 directly interacts with the proline‐rich domain of p53 through its BAG domain. In addition, the current study exhibited that BAG3 promoted degradation of p53 via a calpain‐dependent manner via direct interaction, since mutant BAG3 with BAG deletion had no effect on the stability of p53. The current study demonstrated a loop regulation between p53 and BAG3 under metabolic stress induced by glucose limitation: p53 suppressed BAG3 expression at the transcriptional level via its recruitment to the *BAG3* gene, while BAG3 promoted calpain‐dependent degradation of p53 via direct interact with its protein. Thereby, BAG3 suppression by p53 may constitute a positive adjustment to guarantee p53 accumulation during metabolic stress.

In summary, this study demonstrates the importance of p53‐mediated BAG3 suppression in protection of cells from metabolic stress induced by glucose limitation. BAG3 directly interacts with p53 to promote calpain‐dependent degradation of p53, and thereby, BAG3 suppression liberates p53 and facilitates its accumulation during metabolic stress. The current study provides important insights for understanding the molecular mechanism(s) underlying the p53‐mediated cellular adaptation to metabolic stress. The results from this study thus provide a potential opportunity to develop novel therapeutic strategy to get rid of cancer cells.

## CONFLICT OF INTEREST

The authors declare no conflict of interest.

## AUTHOR'S CONTRIBUTIONS

Jiamei W, Liu b, Li C and Sun J performed all molecular biology and imaging experiments. Jiang J and Yan J performed MEF isolation and identification. Wang H,Jiamei W and Du Z designed the experiments and wrote the manuscript.

## Data Availability

The data that support the findings of this study are available from the corresponding author upon request.

## References

[1] Hirayama A , Kami K , Sugimoto M , et al. Quantitative metabolome profiling of colon and stomach cancer microenvironment by capillary electrophoresis time‐of‐flight mass spectrometry. Can Res. 2009;69(11):4918‐4925. 10.1158/0008-5472.CAN-08-4806 19458066

[2] Cairns RA , Harris IS , Mak TW . Regulation of cancer cell metabolism. Nat Rev Cancer. 2011;11(2):85‐95. 21258394 10.1038/nrc2981

[3] Schulze A , Harris AL . How cancer metabolism is tuned for proliferation and vulnerable to disruption. Nature. 2012;491(7424):364‐373. 23151579 10.1038/nature11706

[4] Itahana Y , Itahana K . Emerging roles of p53 family members in glucose metabolism. Int J Mol Sci. 2018, 19(3):E776. 29518025 10.3390/ijms19030776 PMC5877637

[5] Jones RG , Plas DR , Kubek S , et al. AMP‐activated protein kinase induces a p53‐dependent metabolic checkpoint. Mol Cell. 2005;18(3):283‐293. 15866171 10.1016/j.molcel.2005.03.027

[6] Sinthupibulyakit C , Ittarat W , St Clair WH , St Clair DK . p53 Protects lung cancer cells against metabolic stress. Int J Oncol. 2010;37(6):1575‐1581. 21042727 10.3892/ijo_00000811 PMC3086553

[7] Takayama S , Xie Z , Reed JC . An evolutionarily conserved family of Hsp70/Hsc70 molecular chaperone regulators. J Biol Chem. 1999;274(2):781‐786. 9873016 10.1074/jbc.274.2.781

[8] Yan J , Liu C , Jiang JY , et al. BAG3 promotes proliferation of ovarian cancer cells via post‐transcriptional regulation of Skp2 expression. Biochim Biophys Acta Mol Cell Res. 2017;1864(10):1668‐1678. 28624440 10.1016/j.bbamcr.2017.06.004

[9] Rosati A , Graziano V , De Laurenzi V , Pascale M , Turco MC . BAG3: a multifaceted protein that regulates major cell pathways. Cell Death Dis. 2011;2:e141. 21472004 10.1038/cddis.2011.24 PMC3122056

[10] Behl C . Breaking BAG: The Co‐Chaperone BAG3 in health and disease. Trends Pharmacol Sci. 2016;37:672‐688. 27162137 10.1016/j.tips.2016.04.007

[11] Gamerdinger M , Hajieva P , Kaya AM , Wolfrum U , Hartl FU , Behl C . Protein quality control during aging involves recruitment of the macroautophagy pathway by BAG3. EMBO J. 2009;28(7):889‐901. 19229298 10.1038/emboj.2009.29 PMC2647772

[12] Gentilella A , Khalili K . BAG3 expression is sustained by FGF2 in neural progenitor cells and impacts cell proliferation. Cell Cycle. 2010;9(20):4245‐4247. 20962586 10.4161/cc.9.20.13517 PMC3055207

[13] Basile A , Pascale M , Franceschelli S , et al. Matrine modulates HSC70 levels and rescues DeltaF508‐CFTR. J Cell Physiol. 2012;227(9):3317‐3323. 22170045 10.1002/jcp.24028

[14] Lee MY , Kim SY , Shin SL , et al. Reactive astrocytes express bis, a bcl‐2‐binding protein, after transient forebrain ischemia. Exp Neurol. 2002;175(2):338‐346. 12061864 10.1006/exnr.2002.7903

[15] Chen L , Wu W , Dentchev T , et al. Light damage induced changes in mouse retinal gene expression. Exp Eye Res. 2004;79(2):239‐247. 15325571 10.1016/j.exer.2004.05.002

[16] Takayama S , Reed JC . Molecular chaperone targeting and regulation by BAG family proteins. Nat Cell Biol. 2001;3(10):E237‐E241. 11584289 10.1038/ncb1001-e237

[17] Doong H , Vrailas A , Kohn EC . What's in the 'BAG'?–A functional domain analysis of the BAG‐family proteins. Cancer Lett. 2002;188(1–2):25‐32. 12406544 10.1016/s0304-3835(02)00456-1

[18] Wang HQ , Liu HM , Zhang HY , Guan Y , Du ZX . Transcriptional upregulation of BAG3 upon proteasome inhibition. Biochem Biophys Res Comm. 2008;365(2):381‐385. 17996194 10.1016/j.bbrc.2007.11.001

[19] Pagliuca MG , Lerose R , Cigliano S , Leone A . Regulation by heavy metals and temperature of the human BAG‐3 gene, a modulator of Hsp70 activity. FEBS Lett. 2003;541(1–3):11‐15. 12706811 10.1016/s0014-5793(03)00274-6

[20] Tabuchi Y , Ando H , Takasaki I , et al. Identification of genes responsive to low intensity pulsed ultrasound in a human leukemia cell line Molt‐4. Cancer Lett. 2007;246(1–2):149‐156. 16678341 10.1016/j.canlet.2006.02.011

[21] Lee MY , Kim SY , Choi JS , et al. Induction of Bis, a Bcl‐2‐binding protein, in reactive astrocytes of the rat hippocampus following kainic acid‐induced seizure. Exp Mol Med. 2002;34(2):167‐171. 12085992 10.1038/emm.2002.24

[22] An MX , Li S , Yao HB , et al. BAG3 directly stabilizes Hexokinase 2 mRNA and promotes aerobic glycolysis in pancreatic cancer cells. J Cell Biol. 2017;216(12):4091‐4105. 29114069 10.1083/jcb.201701064 PMC5716268

[23] Matoba S , Kang JG , Patino WD , et al. p53 regulates mitochondrial respiration. Science. 2006;312(5780):1650‐1653. 16728594 10.1126/science.1126863

[24] Bensaad K , Tsuruta A , Selak MA , et al. TIGAR, a p53‐inducible regulator of glycolysis and apoptosis. Cell. 2006;126(1):107‐120. 16839880 10.1016/j.cell.2006.05.036

[25] Jiang P , Du W , Wang X , et al. p53 regulates biosynthesis through direct inactivation of glucose‐6‐phosphate dehydrogenase. Nat Cell Biol. 2011;13(3):310‐316. 21336310 10.1038/ncb2172 PMC3110666

[26] Hu W , Zhang C , Wu R , Sun Y , Levine A , Feng Z . Glutaminase 2, a novel p53 target gene regulating energy metabolism and antioxidant function. Proc Natl Acad Sci USA. 2010;107(16):7455‐7460. 20378837 10.1073/pnas.1001006107 PMC2867677

[27] Li N , Du ZX , Zong ZH , et al. PKCdelta‐mediated phosphorylation of BAG3 at Ser187 site induces epithelial‐mesenchymal transition and enhances invasiveness in thyroid cancer FRO cells. Oncogene. 2013;32(38):4539‐4548. 23108398 10.1038/onc.2012.466

